# Multicentric Dentigerous Dermoid Cyst with an Unusual Location in the Central Nervous System

**DOI:** 10.1055/s-0036-1583205

**Published:** 2016-04-20

**Authors:** Abidin Murat Geyik, Sırma Geyik, Ibrahim Erkutlu, Mehmet Alptekin, Inan Gezgin, Mehmet Dokur

**Affiliations:** 1 Department of Neurosurgery, Gaziantep University, Gaziantep, Turkey; 2 Department of Neurology, Gaziantep University, Gaziantep, Turkey; 3 Department of Neurosurgery, Kafkas University, Kars, Turkey; 4 Department of Emergency Medicine, Zirve University, Gaziantep, Turkey

**Keywords:** dentigerous dermoid cyst, multicentric localization, central nervous system

## Abstract

Dermoid cysts are benign and congenital ectodermal inclusions. Their occurrence in an intracranial location is quite rare. They constitute 0.1 to 0.7% of all intracranial tumors. Their occurrence in the fourth ventricle and their multicentric feature are extraordinary. A 12-year-old boy was admitted to our clinic with a dermoid cyst with sixth cranial nerve involvement. He had symptoms of increased intracranial pressure. This case is the first dermoid cyst in the literature at this unusual location; a mature tooth structure was found within the cyst, which extended over the cervical subsegments. There was a second dermoid cyst in the thoracic spine (multicentric). Our aim is to present an atypical dermoid cyst along with radiodiagnostic characteristics and macroscopic findings.

In the embryologic period, dermoid cysts originate from the ectodermal cells within the intracranial area. They are benign and slow-growing congenital ectodermal inclusion cysts that rarely undergo malignant transformations. Intracranial dermoid cysts are rarely seen. They constitute less than 1% of head and neck tumors. 1 They are mostly located in the midline, and they can present with various clinical manifestations such as seizures, focal neurologic deficits, meningitis, hydrocephalus, sudden death, or incidental diagnosis. 2 3 In this case report, we present a patient with a cyst starting from the fourth ventricle and extending over the lower cervical segments. The cyst contained a mature tooth and extended into the thoracic spine along with a different lesion of the same nature as evidenced by magnetic resonance imaging (MRI), computed tomography (CT), and macroscopic findings.

## Case Report

**Fig. 1:**
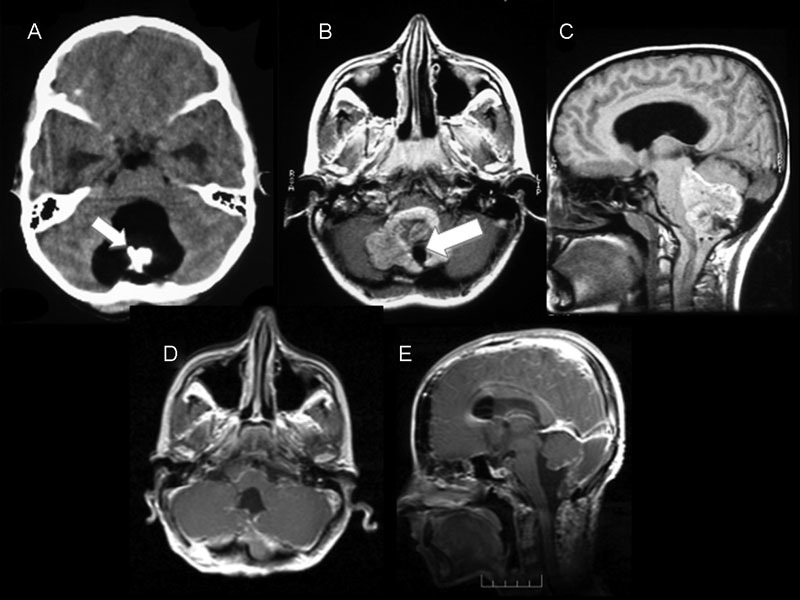
Preoperative and first postoperative radiologic studies of the patient. Axial computed tomography of the brain (A) showing a solid hypodense lesion situated in the fourth ventricle cavity containing a hyperdense bonelike area compared with the cerebral parenchyma in the center (small arrow). Magnetic resonance imaging (MRI) in (B) axial and (C) sagittal sections demonstrating a solid tumor hyperintense in T1-weighted imaging containing a hypointense core located in the fourth ventricle (large arrow). Postoperatively, (D) axial and (E) sagittal T1-weighted MRI with contrast medium showed the gross total resection of the lesion.

**Fig. 2:**
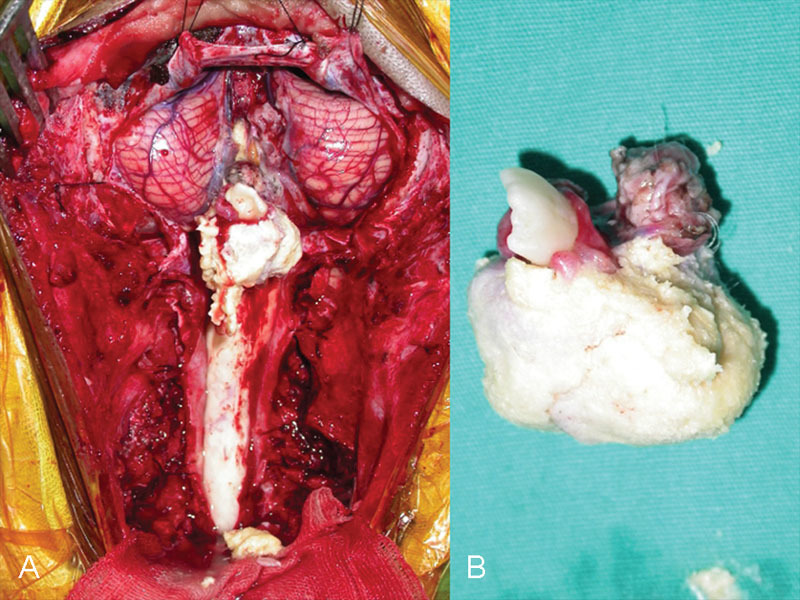
Intraoperative photographs of the lesion. After suboccipital craniotomy and C1–C6 laminectomy, the lesion extending from fourth ventricle cavity to the lower cervical region is seen (A). The mature tooth, its gingiva, and dermoid cyst components such as keratinous structures and hair tufts are seen (B).

A 12-year-old boy had 2-year-long intermittent headache and 10-day-long double vision. His neurologic examination revealed no pathology except for limited abduction in the right eye (right sixth cranial nerve paralysis). His blood biochemistry, complete blood cell count, sedimentation, and C-reactive protein values were normal. The patient's cranial CT showed a hyperdense mass, which had calcified areas within the fourth ventricle ( Fig. 1A ). The cranial MRI performed directly after that showed a soft tissue mass of 4 × 5.5 cm without significant contrast involvement. The mass obliterated the fourth ventricle and compressed the vermis, extending over the level of quadrigeminal cistern, compressing the cerebellum hemisphere in the adjacent brain stem. The mass was hyperintense in T1-weighted images; its fat density and inner areas matched with the observed calcifications ( Fig. 1B, C ). There was a hydrocephalus secondary to the mass compression. In addition to that, cerebellar tonsils were observed as caudal herniation of 4 cm due to the mass effect. On the other hand, in the periventricular area, increased signals matching with T2-weighted fluid-attenuated inversion recovery (FLAIR) hyperintense transependymal resorption were observed. Median suboccipital craniotomy and C1–C2 laminectomy were applied to the patient. Then the dura was opened with a Y incision. After seeing that the mass extended downward, the laminectomy area was also extended over the T2 level ( Fig. 2 ). The soft, dirty yellow mass, which contained a tooth and hair structures including a gingiva component, was totally resected with a microscope, bipolar cautery, and cavitron ultrasonic surgical aspirator.

**Fig. 3:**
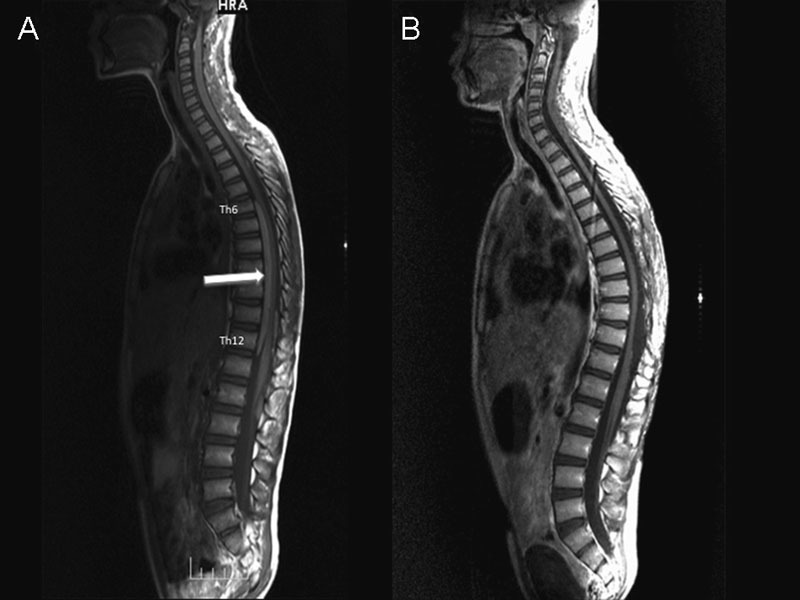
Second postoperative radiologic images. After the first operation, another lesion extending from the sixth to twelfth vertebrae was noticed (A, arrow), and the lesion could be resected totally (B).

**Fig. 4:**
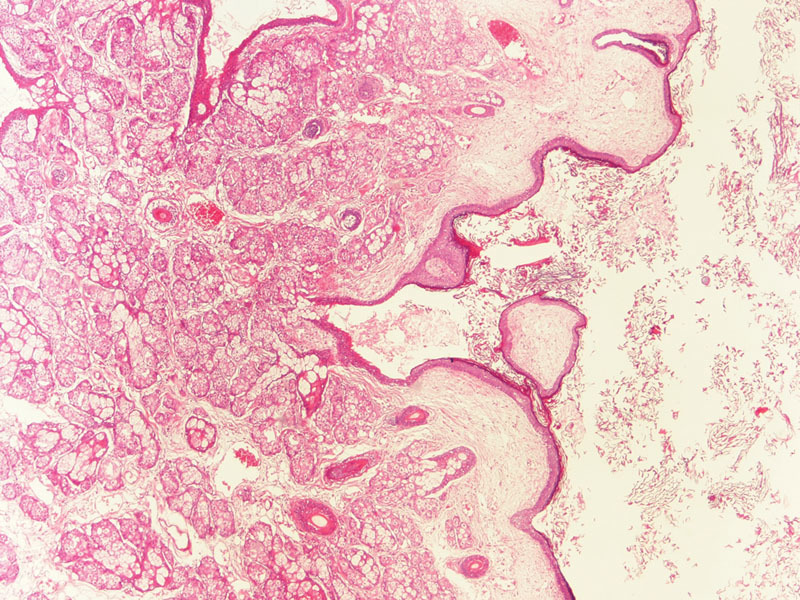
Histopathologic microphotograph. Microscopic specimen of the lesion is compatible with dermoid cyst that is lined by squamous epithelium and endowed with skin appendages, including pilosebaceous units.

After the operation, the patient's clinical symptoms improved. In the control cranial MRI, some postoperative changes were observed ( Fig. 1D, E ). However, on the postoperative thoracic MRI, nonopacifying areas, of which the biggest was extramedullary located at the T6–T12 level (with an approximate size of 13 × 6 mm), were viewed as hyperintense in T2-weighted images and as slightly hyperintense in T1-weighted images with intravenous contrast medium injection. The described lesions minimally compressed on the spinal cord ( Fig. 3A ). The histopathologic study confirmed the diagnosis of dermoid cyst ( Fig. 4 ). In the second postoperative month, the patient was operated on again due to the thoracic lesion. Hemilaminectomy was applied on T6, T7, T8, T9, T11, and T12 vertebrae from the right side by a high-speed drill. The mass, which was located anterior and lateral to the cord and around the dorsal root in the subarachnoid space, was removed totally. The pathologic results were similar to the results of the mass formerly removed from the cranium. The postoperative thoracic MRI showed that the mass was totally resected ( Fig. 3B ). No complications occurred in the patient after the two surgeries, and he was discharged from the hospital after the usual neurologic examination.

## Discussion

Intracranial dermoid cysts are rarely seen cystic benign tumors that may contain hair, sweat, and fat components generated by the epidermis and dermis. They constitute 0.04 to 0.7% of the intracranial tumors. 1 4 They are mostly seen in the pediatric age group. Although the location of dermoid cysts in the posterior fossa is not usual, they are located mostly in the midline or around the midline; the location can also occur in the fourth ventricle. 5 6 Logue and Till classified posterior fossa dermoid cysts into four groups, depending on whether they are extradural or intradural, and on the degree of development of the dermal sinus, whether absent, partial, or complete. 7 Infratentorial dermoids are more frequently seen in the first two decades of life. In the article by Orakcioglu et al, only 1 of 7 patients had posterior fossa location. 3 Based on the location of the cysts, clinical manifestations can include headache, dizziness, ataxia, cranial nerve paralysis (mostly sixth or seventh cranial nerve), papilledema, seizure, and aseptic meningitis due to the spread of cyst contents. However, the clinical course was based on the increased intracranial pressure and hydrocephalus induced by mass effect. 8 Our 12-year-old patient was admitted to the hospital with diplopia related to the cranial nerve involvement, and cranial CT and MRI showed a mass within the fourth ventricle causing hydrocephalus. No dermal sign was seen in his examination. Some different radiologic features of the dermoid cysts were defined in the literature.

The literature refers to dermoids mostly associated with hyperintensity in T1-weighted images and hypointensity in T2-weighted images, no suppression in FLAIR image, and intermediate restriction in diffusion-weighted images due to a higher density of cholesterol within the tumor. 3 9 10 In our case, the CT image showed a hyperdensity with calcifications around the fourth ventricle, and the cranial MRI showed a soft tissue mass without significant contrast involvement, which obliterated the fourth ventricle and vermis in the middle zone of the cerebellum, compressing the cerebellar hemisphere in the adjacent brain stem, which was hyperintense in T1-weighted images; the fat density and inner part matched the calcifications. These images matched the descriptions in the literature, and the density of the calcification within the lesion was remarkable. During the operation, although the mass extending over the lower cervical levels was unexpected, it was a mistake that a preoperative cervical image was not taken. On the control thoracic MRIs performed after that, the second lesion of same nature located at T6–T12 level, which was not in continuity with the first lesion, led us to consider multifocal location.

Our opinion was confirmed with the same pathology results after the second operation. Multifocal location of dermoid cysts is quite rare. 11 The mature tooth removed from our case had not been seen before in the dermoid cysts located in the fourth ventricle or other dermoid cysts with posterior fossa locations mentioned in the literature. 1 7 8 12
